# Radiation Resistance of High-Entropy Alloys CoCrFeNi and CoCrFeMnNi, Sequentially Irradiated with Kr and He Ions

**DOI:** 10.3390/ma17194751

**Published:** 2024-09-27

**Authors:** Bauyrzhan Amanzhulov, Igor Ivanov, Vladimir Uglov, Sergey Zlotski, Azamat Ryskulov, Alisher Kurakhmedov, Asset Sapar, Yerulan Ungarbayev, Mikhail Koloberdin, Maxim Zdorovets

**Affiliations:** 1 Institute of Nuclear Physics, Almaty 050032, Kazakhstan; igor.ivanov.inp@gmail.com (I.I.); ryskulov_nbd@mail.ru (A.R.); kurahmedov.alisher@gmail.com (A.K.); koloberdin@inp.kz (M.K.);; 2 Physical-Technical Faculty, L.N. Gumilyov Eurasian National University, Astana 010008, Kazakhstan; 3 Engineering Profile Laboratory, L.N. Gumilyov Eurasian National University, Astana 010008, Kazakhstan; 4 Department of Solid State Physics, Belarusian State University, 220030 Minsk, Belarus; uglov@bsu.by (V.U.); zlotski@bsu.by (S.Z.)

**Keywords:** high-entropy alloys, radiation resistance, GIXRD analysis, macrostress, dislocation density

## Abstract

This work studied the effect of sequential irradiation by krypton and helium ions at room temperature on the composition and structure of CoCrFeNi and CoCrFeMnNi high-entropy alloys (HEAs). Irradiation of the HEAs by 280 keV Kr^14+^ ions up to a fluence of 5 × 10^15^ cm^–2^ and 40 keV He^2+^ ions up to a fluence of 2 × 10^17^ cm^–2^ did not alter their elemental distribution and constituent phases. Blisters formed on the nickel surface after sequential irradiation, where large blisters had an average diameter of 3.8 μm. The lattice parameter of the (Co, Cr, Fe and Ni) and (Co, Cr, Fe, Mn and Ni) solid solutions increased by 0.17% and 0.37% after sequential irradiation, respectively. Irradiation by Kr ions led to a decrease in tensile macrostresses in the HEAs in the region of krypton ion implantation (Region I) and the formation of compressive macrostresses in the region behind the peak of implanted krypton (Region II). Sequential irradiation formed large compressive stresses in Ni and HEAs equal to −131.5 MPa, −300 MPa and −613.5 MPa in Ni, CoCrFeNi and CoCrFeMnNi, respectively, in the Region II. Irradiation by krypton ions decreased the dislocation density by 1.6–2.3 times, and irradiation with helium ions increased it by 11–15 times relative to unirradiated samples for CoCrFeNi and CoCrFeMnNi, respectively. Sequentially irradiated CoCrFeMnNi HEA had higher macrostresses and dislocation density than CoCrFeNi.

## 1. Introduction

The development of new generation-IV reactors with larger power includes the operation at higher temperatures and radiation damage. Structural materials for such reactors need to operate at temperatures of 500–800 °C and higher and withstand radiational damage of around 100–200 displacements-per-atom (dpa) [1,2] without degradation of their mechanical properties. Conventional ferritic-martensitic steels suffered from embrittlement, and austenitic steels had low resistance to void swelling [1]. Ni-based FCC high-entropy alloys (HEAs) have demonstrated comparable or enhanced resistance to radiation-induced segregation, swelling compared to the steels used as structural materials [3]. HEA research can also bring benefit to the elements of aerospace engineering [4]. Although Co and Ni are considered high activation elements [3], the knowledge of how such Ni-based HEAs resist radiation-induced degradation can be useful for similar HEAs less susceptible to neutron activation.

High-entropy alloy is usually defined as an alloy of at least five main elements with equal or close to equal concentrations of elements, where each main element’s content is in the range of 5–35 atomic percents (at.%) and whose configurational entropy is Δ*S_conf_* ≥ 1.5*R* (where *R* is a universal gas constant) [5,6]. Elevated entropy of mixing of HEAs enables the creation of thermodynamically stable simple solid solutions and suppresses segregation [3]. HEAs have high formation energy of point defects, low defect mobility, high atomic-level stresses and distinct defect recovery mechanisms due to these intrinsic properties [7,8,9].

Analyzing the irradiation effects of combined heavy and light ions is important for understanding the structural changes in reactor materials, which are subject to irradiation by neutrons, fission products and transmutation products. Heavy ions can be used to simulate the irradiation by fission products and neutrons, have a higher dose rate, require a shorter irradiation duration for large damage generation, produce large clusters of defects and have more pronounced damage peaks and damage varying continuously over depth [10]. Light ions produce isolated point Frenkel pairs and small clusters and have larger penetration depth. Helium is produced during transmutation reactions, is to a large extent insoluble in metals and has higher implantation rate than krypton [10]. The He migration regime and bubble formation depend on the irradiation temperature, initial (pre-existing defects) and irradiation-induced defects. At low temperature (<0.2 *T_m_*, where *T_m_* is the melting temperature of a material) irradiation, He clustering is based on athermal processes, where He migrates by collisions and direct atomic displacements or by migration through interstitial sites before being trapped by a defect [11]. Helium atoms also have a strong tendency to form clusters with vacancies if a vacancy concentration is sufficient [3]. He bubbles can grow, aggregate to form blisters and cause surface exfoliation [10,12].

Krypton ion irradiation produces damage in HEAs, but Ni-based HEAs tend to preserve a stable single phase. Irradiation of NiCoFeCr and NiCoFeCrMn HEAs with 1 MeV Kr ions at 773 K to 2 dpa led to the formation of dislocation loops, which were smaller in HEAs than in Ni [13]. After the irradiation of CoCrFeMnNi and 316 H steel by 1 MeV Kr at 500 °C to 1 dpa, no phase transformations were observed, and irradiation-induced hardening was lower in CoCrFeMnNi than in 316 H, but the HEA had a larger dislocation loop size and smaller density of loops [14]. NiCoFeCr, NiCoFeCrMn HEAs irradiated by He ions at 450–700 °C had larger density but a smaller diameter of He bubbles than Ni or steel 304 [15,16,17].

Many studies were carried out on high temperature and near-melting point irradiation of samples. But low-temperature effects are also important as reactor components operate at a range of temperatures. For example, irradiation by krypton with an energy 1 MeV at a low temperature of 300 °C up to 1 dpa caused similar hardening and high density of dislocation loops in both FCC 316 H steel and CoCrFeMnNi, and it was argued that at this temperature, microstructural evolution is less dependent on composition and configurational entropy, which can be due to restricted long-range diffusion [18]. When irradiated with He of 90 keV energy at room temperature, Fe-Co-Ni-Cr had lower density of He bubbles up to 5 × 10^17^ cm^−2^, which is related to slower diffusion and a distorted lattice more effectively trapping He atoms, and at 1 × 10^18^ cm^−2^, blisters smaller than 10 μm started to form in Fe-Co-Ni-Cr, while larger blisters and exfoliation were observed in Ni [19].

Sequential irradiation with Kr and He leads to an increase in damage and facilitates He bubble formation. After dual-beam irradiation of Nickel with 16 keV He and 1 MeV Kr at 500 °C, He bubbles had a homogeneous distribution and a smaller size compared to single-beam He irradiation [20]. Another structural material, the SiC composite pre-implanted with He, was sequentially irradiated by 800 keV Kr and 50 keV He ions up to 4–16 dpa at 1073 K [21]. It was proposed that in sequential irradiation, bubble growth was driven by Kr irradiation and vacancy production, but then it was weakened by re-dissolution He atoms in bubbles, probably caused by collision cascades and thermal spikes [21]. Sequential irradiation of LiNbO_3_ with Fe^+^ and He^+^ resulted in the superposition of damage peaks from Fe near the surface and a long peak from He, and a deeper and long-range strain was formed [22].

FCC Ni-based HEAs have relatively high radiation resistance compared to conventional steels and some nickel alloys [3,8,16], but the combined effects of heavy and light particles irradiation on these HEAs still require studying to approach closer-to-reactor irradiation simulation. This work concentrated on investigating the radiation resistance of CoCrFeNi and CoCrFeMnNi HEAs after sequential irradiation by Kr and He ions at room temperature, as well as establishing the mechanisms of radiation defects’ behavior, which is a continuation of work on researching He irradiation influence on the HEAs [23].

## 2. Materials and Methods

CoCrFeNi, CoCrFeMnNi and nickel samples were produced at the Beijing Institute of Technology (Beijing, China) by the procedure described in our previous study [23]. Ingots of HEAs and Ni were obtained from the powders of metals (purity reaching 99.97%) by arc melting in the atmosphere of high-purity argon and casting into a copper mold. After their crystallization, the ingots were annealed for 24 h at 1150 °C to spheroidize and homogenize the grain structure of the samples. Ingots were cold-rolled to reduce the thickness by 85% and at the end, annealed at 1150 °C for 72 h to decrease the amount of texture and stresses caused by rolling. HEA and nickel samples had the shape of rectangular parallelepipeds with linear dimensions of 5.0 mm × 5.0 mm × 1.5 mm [23].

Irradiation of the samples was performed in a DC-60 heavy ion accelerator located in the Astana branch of the Institute of Nuclear Physics (Astana, Kazakhstan). HEAs and nickel samples were first irradiated by 280 keV Kr^14+^ ions up to a fluence of 5 × 10^15^ cm^–2^ and then by 40 keV He^2+^ ions up to a fluence of ions 2 × 10^17^ cm^–2^ at room temperature. Krypton and helium ion irradiation parameters are provided in Table 1.

The morphology of the surface and elemental composition of the samples were analyzed by scanning electron microscopy (SEM) and energy-dispersive X-ray spectroscopy (EDX) methods on a Hitachi TM3030 microscope (Hitachi, Tokyo, Japan) at an accelerating voltage of 15 kV. Blister diameters were measured from SEM images using ImageJ software (version 1.54d) [24] and plotted as histograms.

The distribution of elements by depth in CoCrFeNi and CoCrFeMnNi HEAs was also studied in the DC-60 cyclotron by the Heavy Ion Rutherford Backscattering Spectrometry (HIRBS) method. HIRBS can be used to conduct fast, non-destructive analysis, with high mass and depth resolution. A 14 MeV ^14^N^2+^ ion beam was used for HIRBS measurements. A Au–Si semiconductor surface barrier detector with an energy resolution of ~11 keV placed at a scattering angle of 160° was used to detect backscattered particles.

Phases and structures were studied using X-ray diffraction (XRD) analysis with a Rigaku Ultima IV X-ray diffractometer (Rigaku, Tokyo, Japan). XRD patterns in parallel beam geometry were obtained, utilizing CuKα radiation (λ = 0.154179 nm). The samples were constantly rotated at a rate of 30 rps during an XRD scan to reduce the effect of alloy texture. A small-angle X-ray diffraction (SAXRD) mode at an angle of incidence α of the X-ray beam was used to concentrate on the analysis of the irradiated region of the samples. The internal stresses were calculated by the g-sin^2^ψ method [25], which allows estimating stresses in the layers close to the surface by varying the incidence angle α and the depth of scanning of the X-ray beam. The density of dislocations was calculated by the Williamson–Hall method [26], which allows for the separation of the contribution of coherent scattering regions (CSRs) and microstrains to the broadening of a diffraction peak. The density of dislocations (ρ) was estimated by the equation ρ = 3/D^2^ [27], where D is the CSR size.

## 3. Results and Discussion

### 3.1. Composition and Structure of Initial CoCrFeNi and CoCrFeMnNi HEAs

The results of studying the surface microstructure and elemental contents of initial CoCrFeNi and CoCrFeMnNi HEAs were presented in our previous work [23]. The alloys are solid solutions (Co, Cr, Fe and Ni) and (Co, Cr, Fe, Mn and Ni) with an FCC lattice and a single phase, large grains (80–100 μm) and a homogeneous spread of elements by depth. In CoCrFeNi and CoCrFeMnNi alloys, macrostresses reached 103 ± 10 and 44 ± 5 MPa, respectively, and had a tensile nature, and these macrostresses originated due to mechanical treatment during samples’ synthesis [23].

### 3.2. Composition and Structure of CoCrFeNi and CoCrFeMnNi HEAs, Irradiated by Krypton and Helium Ions

**Figure 1:**
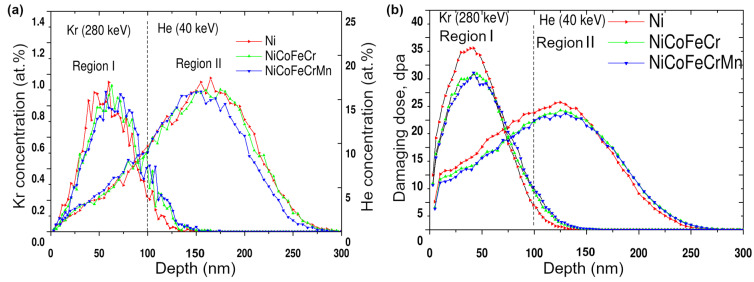
The profile of (**a**) implanted krypton and helium and (**b**) radiation damage in Ni and CoCrFeNi and CoCrFeMnNi HEAs irradiated by Kr^14+^ (280 keV, 5 × 10^15^ cm^−2^) and He^2+^ (40 keV, 2 × 10^17^ cm^−2^).

Figure 1 shows the distribution profiles of implanted krypton and helium and the damaging doses in Ni, CoCrFeNi and CoCrFeMnNi alloys sequentially irradiated by Kr^14+^ (280 keV) and He^2+^ (40 keV) ions. Calculations performed in Stopping and Range of Ions in Matter (SRIM) [28] showed that the projective range of krypton ions in the samples was 59–65 nm and helium 137–146 nm, while maximum Kr ions lost energy equal to 1.1 keV/nm in the region up to 50 nm, and He ions lost 0.22 keV/nm in the region up to 100 nm.

Figure 1 illustrates that the maximum concentration of the implanted ions and radiation-induced damage are approximately 0.95 at.% and 33 dpa, 17.40 at.% and 23.2 dpa for krypton and helium ions, respectively. Implantation profiles of krypton and helium and the damaging doses follow a normal distribution with an asymmetrical shift for helium ions near the surface, which is regular for implantation spreads at ion energies exceeding 10 keV. Helium ions near the surface lose a smaller proportion of energy to nuclear stopping; so, their scattering is reduced.

**Figure 2:**
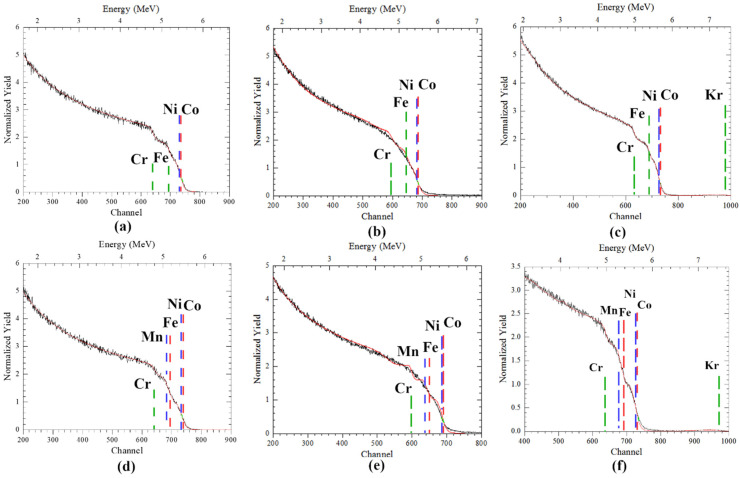
HIRBS spectra (black—experimental, red—RUMP) of: (**a**,**d**) initial [23] and irradiated by (**b**,**e**) Krypton ions, (**c**,**f**) Kr and He ions, (**a**–**c**) CoCrFeNi and (**d**–**f**) CoCrFeMnNi HEAs.

HIRBS and EDX results for elemental concentrations and spatial distribution of elements of initial CoCrFeNi, CoCrFeMnNi and those irradiated with krypton and helium ions are shown in Table 2 and Figure 2. According to Table 2, initial CoCrFeNi and CoCrFeMnNi alloys are nearly equiatomic with 25 and 20 at.% for each element, respectively. Ni and Co, Fe and Mn, have close masses, complicating the accurate calculation of the concentration of individual elements.

Analysis of the spectra obtained by the HIRBS method showed that the initial CoCrFeNi and CoCrFeMnNi alloys possess a uniform spread of elements in depth (Figure 2a,d).

Table 2 shows that in CoCrFeNi and CoCrFeMnNi samples after irradiation with Kr^14+^ and He^2+^ ions, relatively small (less than 6%) concentration variations emerged. In this case, Kr irradiation alone results in a slight increase in the concentration of Fe for the CoCrFeNi HEA and the concentrations of Fe and Ni for the CoCrFeMnNi HEA. The concentrations of the remaining elements decrease. Subsequent helium irradiation raises the concentrations of Co and Ni for the CoCrFeNi HEA and those of Co and Cr for the CoCrFeMnNi HEA. Relatively large variations in the concentration of elements in the samples after irradiation could be due to the migration of radiation-induced vacancies and their aggregation with helium atoms as well as the features of the HIRBS technique.

Nevertheless, irradiated alloys preserve their near-equiatomic concentrations, which is mostly homogeneous apart from small redistribution of elements in the penetration layer of krypton and helium ions throughout the depth measured by HIRBS (Figure 2), and thus HEAs seem to successfully withstand radiation-stimulated segregation. In addition, Kr implantation was taken into account, but it was relatively low compared to helium.

After sequential irradiation with Kr and He, steps appear in the RBS spectrum of both CoCrFeNi and CoCrFeMnNi, but the spectra are similar to the initial. The height of steps is proportional to stopping cross-section, scattering cross-section of elements and kinematic factor [29]; therefore, edges of Ni and Co with similar atomic numbers and masses are close on the spectra. Radiation-induced segregation will increase the RBS yield of an element in the higher channel, as was in the VCr alloy [30], but the yield pattern in HEAs after Kr and He sequential irradiation was similar to initial. In the RBS spectra in Figure 2 after Kr and sequential irradiation, no peaks similar to those in the RBS spectra of the Hastealloy after He irradiation [31] or the Ni–Ge–Al alloy, irradiated by He ions at 405–605 °C [32] were found. This can also be due to low-temperature irradiation and similarity of the HEA elements. Averback et.al. [33] proposed that in the Ni–Si alloy, 3.25 MeV high-energy Kr at 580 °C irradiation induces less segregation than He irradiation because of production of defects in a narrower region and stronger recombination of point defects due to displacement cascades, while He irradiation produces point defects by low-energy recoils and spreads across a wider range in depth.

Modification of the composition of the HEAs by krypton ion irradiation could be mainly associated with the vacancy diffusion mechanism and the generation of many vacancies. A slight increase in the concentration of Fe and Ni in the HEAs after Kr irradiation, apparently, occurs due to the migration of Fe atoms to the surface from the depth of the samples by the vacancy exchange mechanism, while Ni atoms aggregate near defect clusters [34,35]. Diffusion of atoms in HEAs is affected by lattice distortion, configuration of d-shell electrons, nearby defect clusters and defects sinks [3,36]. The increase of Ni and Co in CoCrFeNi and Co in CoCrFeMnNi after sequential irradiation could be explained by their aggregation near dislocation loops [35] since the density of dislocations increased after the irradiation in both regions. A decrease in the concentration of Mn in CoCrFeMnNi is apparently associated with the processes of radiation segregation along the grain boundaries [37].

Thus, sequential irradiation with krypton and helium ions at room temperature does not alter the elemental composition of Ni and two HEAs significantly.

**Figure 3:**
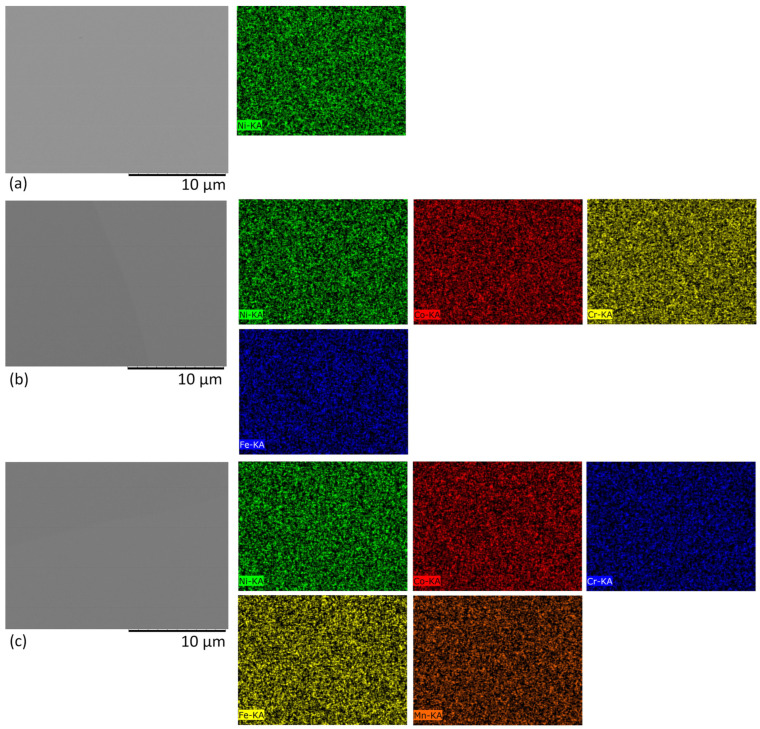
SEM images in the backscattering electrons mode (BSE) of the sample surface irradiated by: (**a**–**c**) Kr ions, where (**a**) Ni, (**b**) CoCrFeNi, (**c**) CoCrFeMnNi and their respective elemental maps.

As shown by Figure 3a–c, after irradiation with low-energy Kr ions, no large defect clusters were found on the surfaces of nickel and HEAs. According to EDS maps, the constituent elements were also uniformly distributed.

**Figure 4:**
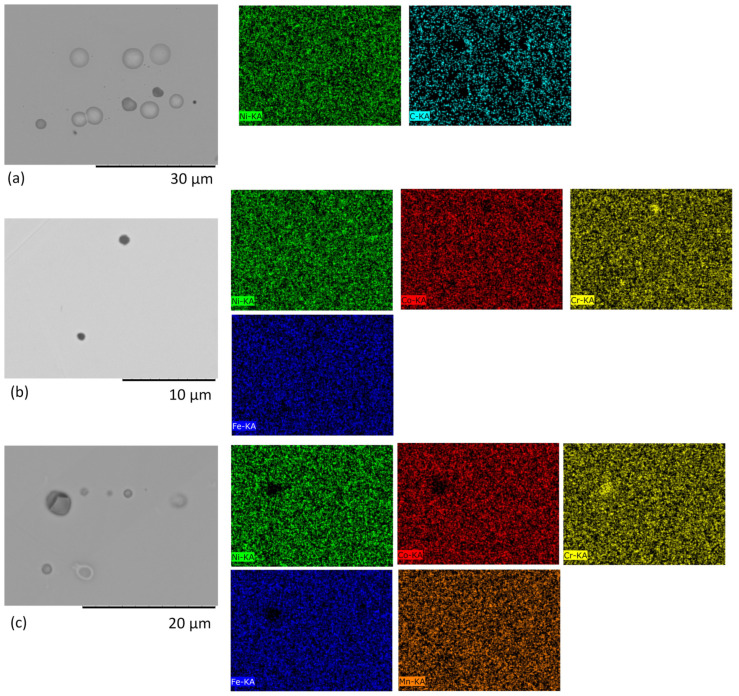
SEM images in the backscattering electrons mode (BSE) of the sample surface irradiated by: (**a**–**c**) Kr and He ions, where (**a**) Ni, (**b**) CoCrFeNi, (**c**) CoCrFeMnNi and their respective elemental maps.

**Figure 5:**
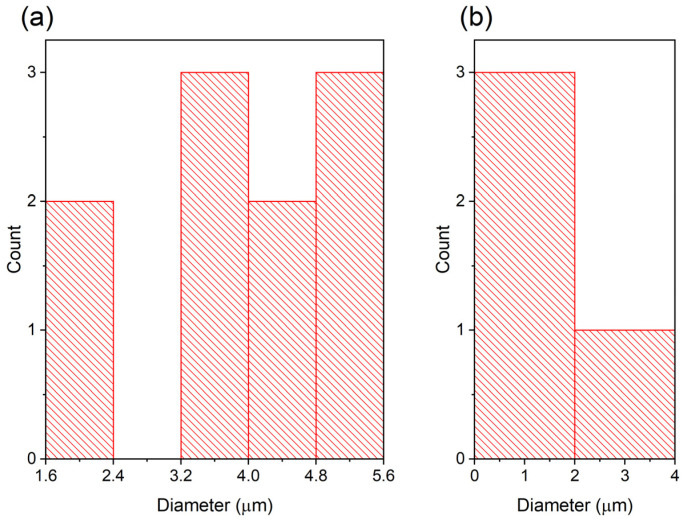
Diameters of blisters from the surface SEM analysis of (**a**) Ni and (**b**) CoCrFeMnNi irradiated by Kr and He ions, corresponding to Figure 4a and Figure 4c, respectively.

But Figure 4a–c illustrates that after irradiation with krypton and helium ions, blisters formed on the surface of Ni (Figure 4a). As can be seen in Figure 4a and Figure 5, closed blisters with an average diameter of 3.8 μm are present on the surface of the nickel samples sequentially irradiated by krypton and helium ions. No clear blisters were observed in CoCrFeNi, but a few round dark defects with sizes of 1–1.5 μm appear, which are poor in Co and rich in Cr. This could be due to local segregation of Cr, which tends to migrate faster than Ni/Co by exchange with vacancies [37], which move towards grain boundaries [3]. In CoCrFeMnNi, several spherical defects resembling blisters less than 1.3 μm in size and a defect of 3.6 μm in size, which is possibly a cracked blister, were observed after sequential irradiation. The dark defects in CoCrFeNi and the cracked defect in CoCrFeMnNi could also be remaining Cr-oxide inclusions found in as-synthesized HEAs [18]. This also agrees with the fact that blisters did not form in both HEAs under He-only irradiation at room temperature [23]; so, only a few blisters might be found in CoCrFeNi and CoCrFeMnNi after room-temperature irradiation even after the damage increase by pre-irradiation with Kr ions. In contrast, many blisters form at high temperatures in both Ni and HEAs [38].

A larger number of large blisters in Ni can be due to more damage produced than in HEAs as estimated by SRIM, which increases the portion of helium-vacancy clusters, leads to the accumulation of He bubbles and facilitates their growth. Observation of blisters in the CoCrFeMnNi HEA could be due to the damage from Kr irradiation supplying vacancies for He bubble formation [20] and thus contributing to blistering. Presence of smaller blisters or their lack in the HEAs is probably related to their lattice distortion and the lower mobility of point defects, which affect the bubble nucleation and growth [19], but no surface exfoliation was observed. A study of the TaTiNbZr HEA irradiated by high-flux He ions at 0–70 eV showed that blister size increases with the increasing energy of ions, and one of the reasons why blisters and microcracks form is the oversaturation of helium and accumulation of He bubbles under the surface [38].

**Figure 6:**
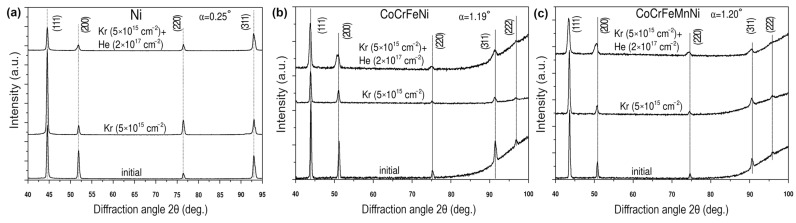
XRD patterns of initial, irradiated by Kr and sequentially irradiated by Kr and He ions samples of: (**a**) Ni, (**b**) CoCrFeNi and (**c**) CoCrFeMnNi HEAs, collected at the X-ray incidence angle α.

Figure 6 presents the phases of Ni and HEAs after irradiation by krypton ions and sequential irradiation by krypton and helium ions. Small-angle XRD patterns were collected at angles of α = 0.25°, 1.19° and 1.20° (Region II) and an X-ray analysis range of 300 nm in nickel, CoCrFeNi and CoCrFeMnNi, respectively.

According to Figure 6, XRD patterns of krypton-irradiated and successively irradiated samples did not have diffraction peaks of new phases and preserved initial (Co, Cr, Fe and Ni) and (Co, Cr, Fe, Mn and Ni) solid solution phases.

It was found that ion irradiation increases the angular position of the diffraction peaks of nickel (Figure 6a) and lowers the angular positions of the diffraction peaks of HEAs’ solid solutions (Figure 6b,c). Calculations showed that irradiation by krypton ions and sequential irradiation by krypton and helium ions caused the lattice shrinking of nickel by 0.16% and 0.15%, respectively; the lattice expansion of the solid solution (Co, Cr, Fe and Ni) by 0.13% and 0.17%, respectively; and the lattice expansion of (Co, Cr, Fe, Mn and Ni) by 0.11% and 0.37%, respectively.

The rising value of the lattice constant of the HEAs is probably a result of the processes of accumulation of point defects and the growth of the number density of helium-vacancy clusters [39]. In addition, in CoCrFeMnNi, lattice expansion exceeded that of CoCrFeNi, which is consistent with a previous work [40], which showed the hindering effect of intrinsic lattice distortion on lattice expansion in HEAs. Nickel’s lattice shrinking could be caused by the He bubble pressure exerted on the lattice atoms to bring them closer [41].

To assess the development of the defect structure of Ni and both HEAs following successive krypton and helium irradiation, studies of macrostresses and dislocation density were carried out in the region preceding the maximum of helium implantation (Region I—up to 100 nm) and in the maximum He implantation region (Region II—from 100 to 300 nm) in Figure 1. Region I also corresponds to the region of krypton ion irradiation and high radiation damage.

**Figure 7:**
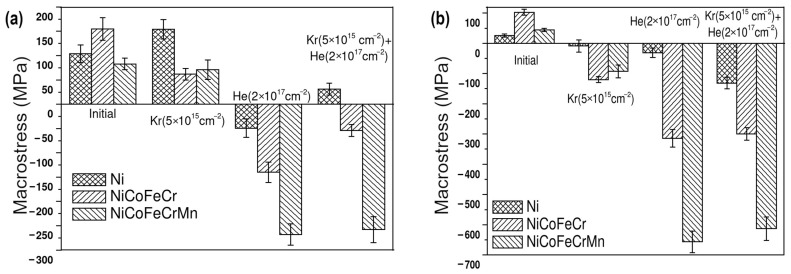
Macrostresses in the initial [23], irradiated by Kr ions, irradiated by He ions [23] and sequentially irradiated by Kr and He ions Ni, CoCrFeNi and CoCrFeMnNi HEAs at XRD scan ranges of: (**a**) 100 nm and (**b**) 300 nm.

The g-sin^2^ψ method was applied to evaluate the macrostresses in Ni and HEAs. Figure 7 displays the macrostresses of as-synthesized and irradiated Ni, CoCrFeNi and CoCrFeMnNi high-entropy alloys at α = 0.084°, 0.39° and 0.39° (Region I) and 0.25°, 1.19° and 1.20° (Region II), determined for the (111) plane. XRD incidence angles α = 0.084° and 0.39° represent a scan range equal to 100 nm.

According to Figure 7a, in Region I, krypton irradiation reduces tensile macrostresses in the HEAs. Tensile macrostresses in CoCrFeNi fall to 62 MPa, and in the CoCrFeMnNi HEA, they decrease insignificantly to 71 MPa. At the same time, for nickel, the level of tensile macrostresses increases to 174 MPa. Sequential irradiation with krypton and helium ions forms compressive macrostresses in CoCrFeNi and CoCrFeMnNi HEAs equal to −54 and −258 MPa, respectively, and in nickel, tensile macrostresses reach 31 MPa. He irradiation [23] forms compressive macrostresses for all samples. In this case, it is expected that sequential irradiation with krypton and helium ions will lead to the formation of compressive macrostresses for all samples, and their level will be close to the sum of the macrostress values obtained during irradiation with krypton and helium ions separately. But Figure 7a shows such a result for Ni and CoCrFeNi. For the CoCrFeMnNi alloy, macrostresses during irradiation with helium ions and successive irradiation with krypton and helium ions coincide within the error. For nickel, sequential irradiation elevates tensile stresses.

The stress change was mainly influenced by radiation damage and He implantation. Irradiation can lead to a lattice parameter decrease because of defect removal, a decrease in residual stress and a reduction in lattice distortion in HEAs or lattice expansion because of the generation of point defects and clusters in their lattice [39,42]. At room temperature, irradiation creates point defects, small defect clusters, dislocation loops in HEAs [3]. An increase in tensile stress of Ni and a decrease in tensile stresses of CoCrFeNi and CoCrFeMnNi in Region I after Kr irradiation could be explained by the radiation-induced point defects in the Ni lattice and the decrease in lattice distortion in both HEAs.

In nickel, sequential Kr and He irradiation produces increased damage but a low He implantation dose in Region I. In addition, point defects and helium atoms migrate more freely in Ni than in the HEAs, form bubbles and make them emerge as blisters on the surface [19,41]. Therefore, tensile stress exceeds compressive stress in Ni Region I.

When irradiated with two ion types successively, stresses in Region I probably decrease due to defect recombination and a decrease in lattice distortion, which is large in the HEAs [39,42]. In CoCrFeNi, the stresses decrease but remain compressive since there is He implantation. In CoCrFeMnNi, irradiation has less effect on the stresses, and they remain compressive, since the lattice accumulates a lot of helium, and the HEA tends to contain smaller bubbles compared to Ni [17].

In Region II, irradiation with krypton ions induces compressive macrostresses for all samples (Figure 7b). In this case, the highest compressive stresses are formed in the CoCrFeNi HEA, amounting to −120 MPa. Sequential irradiation drops compressive stresses in Ni, CoCrFeNi HEA and CoCrFeMnNi to −131.5, −300 and −613.5 MPa, respectively. In this case, only for Ni the stress increases and exceeds the sum of compressive stresses from individual irradiations with krypton and helium ions. For CoCrFeNi and CoCrFeMnNi HEAs, compressive macrostresses in the samples irradiated with helium ions and sequentially irradiated with krypton and helium ions coincide within the error limits.

In Region II, compressive stresses form after irradiation with krypton ions in nickel and HEAs (Figure 7b), while, according to SRIM, the concentration of implanted krypton in this region is about 0.2 at.%, and the damaging dose is less than 3 dpa. A heavy krypton ion produces many point defects in a narrow region near the surface, but some defect clusters can form at depth [3] of Region II, possibly leading to compressive stresses formation [39]. The high concentration of vacancies and helium-vacancy clusters can contribute to the formation of compressive stresses in the HEAs after successive irradiation. It has been already shown [23] that stresses in HEAs sharply increase in deeper Region II compared to near-surface Region I after increasing fluence of He ions, probably caused by increased radiation-induced damage, which can also result from adding Kr irradiation.

As can be seen from Figure 7, the stress in CoCrFeMnNi exceeds that of CoCrFeNi. The reason for the increased stress in CoCrFeMnNi could be a slow defect flow and the accumulation of small defect clusters [3,39].

**Figure 8:**
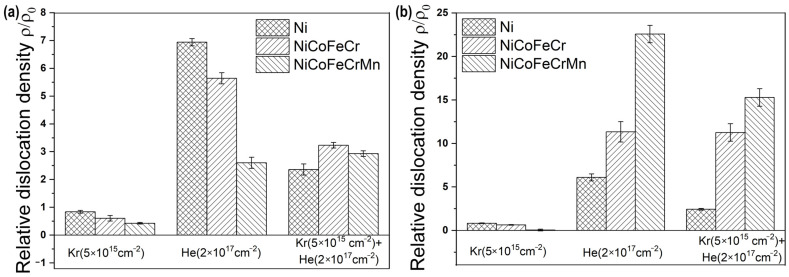
Relative density of dislocations in the initial [23], Kr-irradiated, He-irradiated [23] and sequentially irradiated samples of Ni, CoCrFeNi and CoCrFeMnNi HEAs at an XRD scan range of (**a**) 100 nm and (**b**) 300 nm.

Densities of dislocations in Ni and HEAs for Regions I and II are shown in Figure 8. Irradiation with krypton ions decreases the dislocation density value, and irradiation with helium ions increases it.

Kr irradiation reduces the dislocation density compared to the original samples. In Regions I and II, the density of dislocations in the Ni, CoCrFeNi and CoCrFeMnNi samples decreases by 1.2, 1.6 and 2.3 times, respectively. The density of dislocations in CoCrFeMnNi is higher than that in CoCrFeNi. The density of dislocations in the corresponding nickel and HEAs irradiated with krypton ions in Regions I and II coincides within the error limits.

Sequential irradiation with Kr and He ions substantially elevates the dislocation density in Ni by around 2.4 times for Regions I and II; in CoCrFeNi by 3.2 and 11.3 times for Regions I and II, respectively, and in CoCrFeMnNi by 2.9 and 15.3 times for Regions I and II, respectively (Figure 7). He irradiation considerably raises the dislocation density in all samples, as shown in [23].

The CoCrFeMnNi alloy has a higher dislocation density value in Region II compared to CoCrFeNi.

The relative decrease in dislocation density in both regions during Kr irradiation could be due to a smaller number of point defects, which form dislocations, particularly in HEAs where recombination of vacancies with interstitials is claimed to be stronger [3]. Another reason could be that pre-irradiated dislocations act as point defect sinks as in steel 316 L irradiated by Ne ions at a low temperature of 313K [43]. The small difference between Ni and HEA dislocation density values in Region I after Kr irradiation could be due to limited long-range diffusion of point defects in both Ni and HEAs at a low temperature [18].

Sequential irradiation increases the dislocation densities compared to Kr irradiation because the radiation damage increases in both Regions I and II. The increase of dislocation densities could also be explained by the highly distorted lattice in HEAs which slows the movement of dislocations [40]. In Region II, the dislocation density is higher probably because of helium implantation [22]. In Ni irradiated with a dual beam of Kr and He ions at 500 °C, dislocations also aggregated at a deeper region [20].

Phase composition, macrostress and dislocation density results indicate stronger endurance of the HEAs against successive irradiation by krypton and helium ions compared to Ni.

The elemental content and main phases of CoCrFeNi and CoCrFeMnNi HEAs were preserved despite successive irradiation with krypton (280 keV, 5 × 10^15^ cm^−2^) and helium (40 keV, 2 × 10^17^ cm^−2^) ions; new phases did not emerge, but several blisters formed on the surface of the CoCrFeMnNi HEA, although with a smaller average size than in Ni. Fewer large blisters formed in the HEA probably because of lattice distortion, low defect mobility and weakened helium bubble formation [16,17]. It can be seen that Kr irradiation enhances the surface damage leading to blister formation at room temperature compared to He-only irradiation, which was carried out in [23].

Analysis of the results of the study of macrostresses and density of dislocations in HEAs showed that the main changes obtained as a result of sample irradiation are probably associated with the formation, interaction and migration of radiation-induced defects and their clusters, which modified stresses in the alloys. The compressive stresses in HEAs after irradiation could be caused by the creation of many small point defects, defect clusters [40] and helium bubbles [41], which are constrained by the HEA lattice, while in pure Ni, larger defect clusters form and helium bubbles aggregate into bubbles [19]. The increase in dislocation densities after sequential irradiation could be due to an increased production of point defects compared to Kr-only irradiation, which could contribute to both He bubble formation and dislocations [20].

Higher stress and dislocation density in the CoCrFeMnNi HEA could be caused by its larger lattice distortion [40], high recombination rate and lower He mobility [3], which influence bubble and blister formation. It was shown that in the FeCoNiCr equiatomic alloy, the number density of He bubbles increased from the surface to the peak implanted He region but was still lower than in pure Ni [19]. In addition, to form blisters, bubbles need to grow and overcome the strength of material [44]; therefore, CoCrFeMnNi probably constrained many bubbles at a greater depth [17], and fewer blisters could form on the surface. But the accumulated bubbles and implanted helium beneath the surface could cause lattice strain and a compressive stress increase.

## 4. Conclusions

The arc melting method was used to create bulk high-entropy alloys with FCC solid solutions phases (Co, Cr, Fe and Ni) and (Co, Cr, Fe, Mn and Ni), and a uniform distribution of elements in depth.

It was found that irradiation of CoCrFeNi and CoCrFeMnNi HEAs with Kr^14+^ (280 keV, 5 × 10^15^ cm^−2^) and He^2+^ (40 keV, 2 × 10^17^ cm^−2^) ions does not significantly alter the distribution of elements and phases. But after sequential irradiation, many large blisters with an average diameter of 3.8 μm formed on the Ni surface, while few large blisters formed on the surfaces of the CoCrFeMnNi HEA, and several dark round defects were observed in the CoCrFeNi HEA. Sequential irradiation with krypton and helium ions caused a decrease in the lattice parameter of nickel by 0.15%, lattice expansion of the solid solutions (Co, Cr, Fe and Ni) and (Co, Cr, Fe, Mn and Ni) by 0.17% and 0.37%, respectively.

Krypton ion irradiation reduces tensile stresses in the HEAs and raises tensile stress in the nickel sample in the region of krypton ion implantation (Region I). In the region behind the peak of implanted krypton and maximum implanted helium (Region II), compressive macrostresses formed in Ni and two HEAs due to the diffusion of radiation defects deep into the sample.

Sequential irradiation with Kr and He ions led to a change from tensile to compressive stresses in the HEAs in both Regions I and II compared to unirradiated states. But in Region I, stress in Ni remained tensile, while compressive stresses of −54 MPa and −258 MPa formed in CoCrFeNi and CoCrFeMnNi, respectively. In Region II, compressive macrostresses reached larger values of −131.5 MPa, −300 MPa and −613.5 MPa in Ni, CoCrFeNi and CoCrFeMnNi, respectively. The trend of compressive stress formation similar to He-only irradiation could be due to increased damage and helium bubbles accumulation in the HEAs.

Irradiation by Kr ions led to a decrease in the dislocation density by 1.6–2.3 times in Regions I and II and sequential irradiation with Kr and He ions to an increase of dislocation densities in Region II by 11–15 times for CoCrFeNi and CoCrFeMnNi, respectively.

Most notable macrostress and dislocation density changes were observed in the peak helium implantation region. After irradiation, the CoCrFeMnNi HEA was characterized by higher macrostresses and dislocation density compared to CoCrFeNi.

## Figures and Tables

**Table 1:** Parameters of ion irradiation of Ni, CoCrFeNi and CoCrFeMnNi.

Ions	Energy, keV	Temperature of Irradiation, °C	Average Flux, cm^−2^ s^−1^	Average Irradiation Time	Average Fluence, cm^−2^
Kr^14+^	280	25	1.95 × 10^11^	8 h 30 min	5 × 10^15^
He^2+^	40	25	7.42 × 10^12^	7 h 10 min	2 × 10^17^

**Table 2:** Elemental content (EDX) of CoCrFeNi and CoCrFeMnNi, initial and irradiated at RT.

Sample	Concentration of Elements, at. %				
	Co	Cr	Fe	Mn	Ni
CoCrFeNi (initial)	24.7 ± 0.2	25.7 ± 0.1	25.3 ± 0.1	-	24.3 ± 0.2
CoCrFeNi (Kr^14+^, 5 × 10^15^ cm^−2^)	24.4 ± 0.2	25.4 ± 0.1	26.1 ± 0.1	-	24.1 ± 0.2
CoCrFeNi (Kr^14+^, 5 × 10^15^ cm^−2^) and (He^2+^, 2 × 10^17^ cm^−2^)	25.8 ± 0.2	24.4 ± 0.1	24.9 ± 0.1	-	25.1 ± 0.2
CoCrFeMnNi (initial)	19.5 ± 0.2	20.3 ± 0.1	19.8 ± 0.1	20.6 ± 0.1	19.8 ± 0.2
CoCrFeMnNi (Kr^14+^, 5 × 10^15^ cm^−2^)	18.7 ± 0.2	20.2 ± 0.1	20.2 ± 0.1	20.1 ± 0.1	20.8 ± 0.2
CoCrFeMnNi (Kr^14+^, 5 × 10^15^ cm^−2^) and (He^2+^, 2 × 10^17^ cm^−2^)	20.3 ± 0.2	21.1 ± 0.1	19.4 ± 0.1	20.8 ± 0.1	19.4 ± 0.2

## Data Availability

The original contributions presented in the study are included in the article, further inquiries can be directed to the corresponding author.
